# Imaging the subsurface architecture in porphyry copper deposits using local earthquake tomography

**DOI:** 10.1038/s41598-023-33820-w

**Published:** 2023-04-26

**Authors:** Diana Comte, Gisella Palma, Jimena Vargas, Daniela Calle-Gardella, Matías Peña, Sergio García-Fierro, Joëlle D’Andres, Steven Roecker, Sergio Pichott

**Affiliations:** 1 Departamento de Geofísica, Facultad Ciencias Físicas y Matemáticas, Universidad de Chile, 8370449 Santiago, Chile; 2 Advanced Mining Technology Center, Universidad de Chile, 8370451 Santiago, Chile; 3 Escuela de Geología, Universidad Mayor, Av. Manuel Montt 367, Santiago, Chile; 4 CODELCO, Gerencia de Exploraciones, Casa Matriz, Huérfanos 1270, Santiago, Chile; 5 Research School of Earth Sciences, The Australian National University, Canberra, ACT 2601 Australia; 6 Earth and Environmental Sciences, School of Science, Rensselaer Polytechnic Institute, Troy, NY 12180 USA

**Keywords:** Solid Earth sciences, Geology, Geophysics

## Abstract

An essential part of the world's remaining mineral resources is expected to reside deep in the crust or under post-mineralization cover. For porphyry copper deposits, the world’s primary source of Cu, Mo, and Re, identifying the dynamic processes that control their emplacement in the upper crust can guide future exploration. Seismic tomography can constrain these processes through imaging deep-seated structures at the regional scale. Here we construct a three-dimensional model of the Vp/Vs ratio, based on arrival times of P and S seismic waves, beneath the Cerro Colorado porphyry Cu–(Mo) deposit in northern Chile. Our images show that *low Vp/Vs* (~ 1.55–1.65) anomalies, extending to ~ 5–15 km depth, coincide with the surface expression of known porphyry copper deposits and prospects, as well as delimit structures that host orebodies and related hydrothermal alteration zones. *Medium Vp/Vs* (~ 1.68–1.74) and *high Vp/Vs* (Vp/Vs ~ 1.85) bodies correspond to intermediate-felsic plutonic precursors for porphyry intrusions and mafic magma reservoirs that underlie shallower orebodies, respectively. Imaging these precursor and parental plutons is crucial to the identification of orebodies as they act as the source of fluids for porphyry copper generation. This study demonstrates the potential of local earthquake tomography as a tool to identify future deep mineral resources with minimal environmental impact.

## Introduction

The transition towards a low-carbon future relies on a range of key metals whose demand is expected to substantially increase in the coming decades^1,2^. Porphyry-type deposits are the most important source of Cu, Mo, and Re worldwide^3^, are significant sources of Au and Ag, and might provide significant amounts of other minor and critical metals such as PGEs, REEs, In, Co, Re, Se and Te (e.g., Crespo et al.^4^). Despite their importance for the global supply of a range of metals, the rate of discovery of porphyry copper deposits has steadily decreased in the past decades, since large, shallow, and high-grade deposits have been mostly found and exploited. Brownfield and greenfield discoveries of new orebodies are therefore shifting to greater depth^1^. Exploring deeper orebodies (> 2 km deep) comes with the challenges of identifying the weak “footprints” of deep-seated porphyry copper deposits and the need for new, effective, and unconventional geochemical and geophysical exploration methods^5^.

World-class porphyry copper deposits form mainly along magmatic arcs, above active subduction zones, where they are closely associated with shallow-level intrusive rocks^3,6^. Porphyry copper systems arise from hydrous and oxidized basaltic arc magmas generated in the mantle wedge following the release of fluids and/or hydrous melt from the subducting slab. These basaltic melts differentiate in multi-depth magma reservoirs in the mid-to-lower crust and ascend to the upper crust where they further evolve in large magma chambers, eventually giving rise to evolved, fluid-saturated melts which intrude the shallow crust as plug-like intrusions^3,7,8^. Copper-rich mineralizing fluids are exsolved from these shallow intrusions and released into the surrounding host rock where Cu precipitates as Cu-sulfides.

However, most evolved, shallow crustal intrusions in arc settings are barren, with mineralization being the exception, and despite a good general understanding of porphyry copper systems, the critical conditions for the formation of economic porphyry copper remain poorly understood^7^. This is largely because the parent magmas of porphyry intrusions, the ultimate source of ore-forming fluids, pond in upper crustal magma chambers at 5–15 km depth, several kilometers below the mineralization horizon, where they remain largely inaccessible for direct sampling. A potential means to improve our understanding of the regional scale controls on the formation and emplacement of mineralized porphyry systems is to employ geophysical subsurface imaging methods such as seismic tomography that have not been traditionally implemented in mineral exploration.

Local earthquake tomography is a passive geophysical method that can reveal the architecture of the Earth’s interior through images of seismic wave speeds^9^. This technique uses observations of arrival times from compressional (P) and shear (S) waves produced by local/regional earthquakes and is therefore mostly applied to study the subsurface of seismically active areas such as subduction zones^10^. In recent years, the seismic velocity structure of the mantle and crust, and in particular the ratio of compressional velocity to shear velocity (Vp/Vs), has been shown to be a powerful tool for identifying melt-bearing regions and fluid pathways below active volcanoes^11,12^ and in subduction zone settings^13–15^. Recent studies have also shown a relationship between low Vp/Vs ratios and the location of large ore deposits. Low Vp/Vs anomalies have been identified beneath the supergiant Río Blanco-Los Bronces Cu-Mo porphyry cluster in central Chile^16^, the Olympic Dam IOCG deposit in Australia^17^, the Sorskow Cu-Mo porphyry complex in Russia^18^, the Middle-Lower Yangtze River Metallogenic Belt in China^19^, and the Maricunga Gold Belt in northern Chile^20^. Seismic tomography could therefore become an invaluable asset for the modern exploration of deep exploration targets.

**Figure 1:**
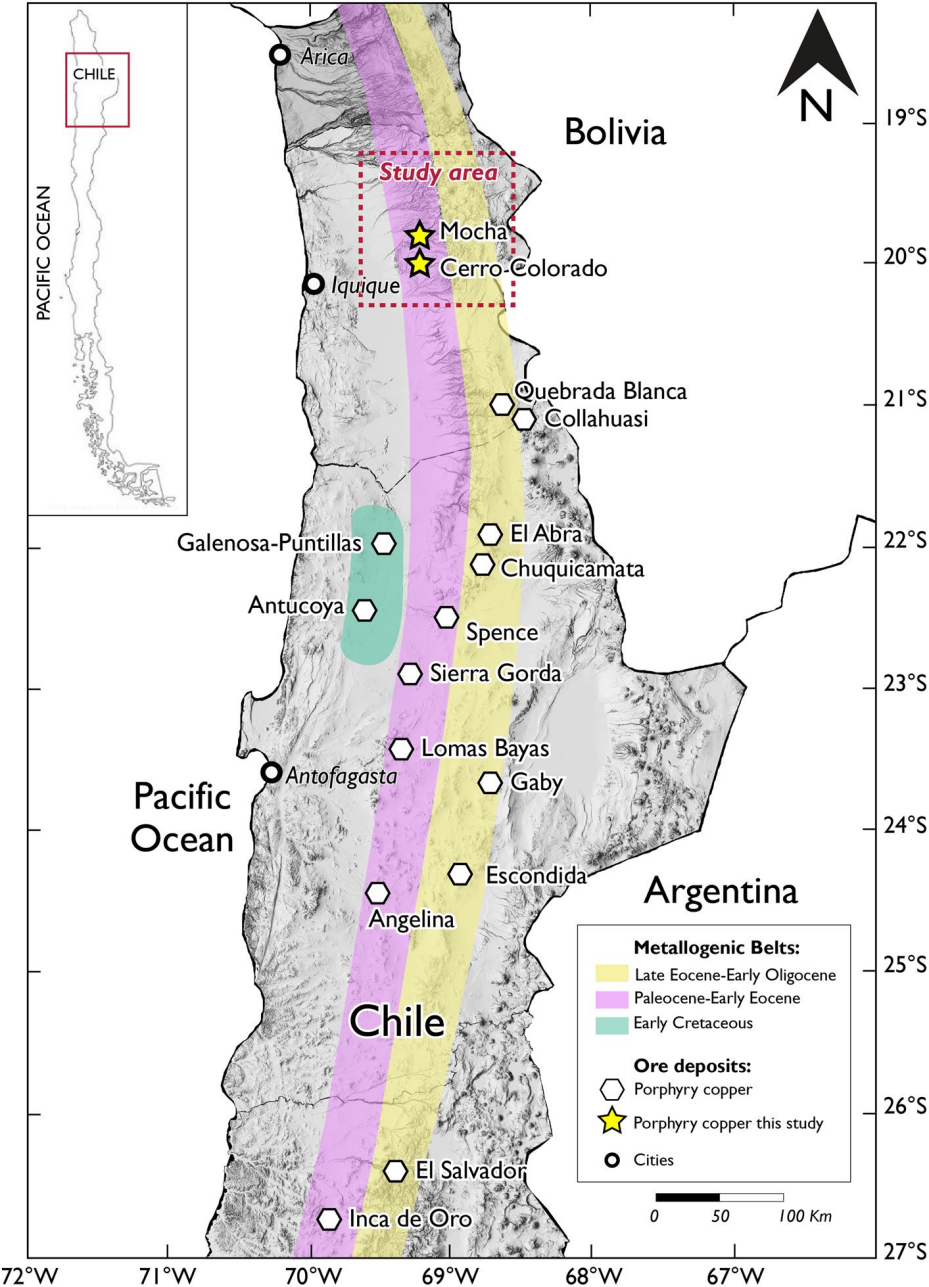
Location of porphyry copper deposits in major metallogenic belts of northern Chile (~ 19.2°S and 20.3°S). The rectangle with red dotted line corresponds to the study area, which includes the Cerro Colorado and Mocha porphyry copper deposits. The distribution of porphyry copper deposits and metallogenic belts are taken from Sillitoe and Perelló^22^. The software used to generate this figure was ArcGIS Pro 10.1 (www.esri.com).

In this study, we use local earthquake tomography to determine the seismic velocity structure of the crust and upper mantle in the vicinity of the Cerro Colorado porphyry copper deposit, and near a range of porphyry prospects (e.g., Mocha) along the Paleocene to Early Eocene and Late Eocene to Early Oligocene copper belts of northern Chile (Fig. 1). We aim to evaluate the use of seismic tomography as a tool for imaging deep-seated structures in porphyry copper systems, including the presence of fluids, intrusive bodies, and hydrothermally altered and mineralized orebodies. We present here a three-dimensional Vp/Vs model of the upper lithosphere above a segment of the northern Chilean subduction zone constructed from 204,943 P- and S-wave arrival times of seismic events recorded by 51 seismic stations between October 9, 2018, and June 28, 2019. Our case study shows that local earthquake tomography, combined with well-established geological models, can be a powerful tool for the exploration of porphyry copper deposits due to its genetic and spatial relation with intrusive bodies, represented by low Vp/Vs anomalies, i.e., high rigidity rocks.

## Study area

The east-dipping subduction of the Farallon and Nazca plates beneath the South American continent from the Mid to Late Jurassic onward has led to orogenesis, arc magmatism and porphyry copper formation in the Andes^21^. Porphyry copper deposits are aligned in arc-parallel belts along the western South American Andean margin, with each belt corresponding to a distinct metallogenic epoch^22^. In northern Chile, porphyry mineralization is associated with two main pulses of magmatic activity from the Paleocene to Early Eocene and from the Late Eocene to Early Oligocene^3,22^. Even though the Paleocene-Eocene copper belt is economically less relevant than the younger, eastern, Eocene–Oligocene copper belt associated with the N-S trending Domeyko fault system (Fig. 1), it hosts a number of large deposits in northern Chile, such as Spence and Cerro Colorado (Fig. 1), and becomes the dominant copper province in southern Peru^23^. Recently, the large distance between deposits in the Paleocene-Eocene copper belt has fueled a search for concealed deposits to the north of Mocha and to the south of Cerro Colorado.

### Geologic setting

**Figure 2:**
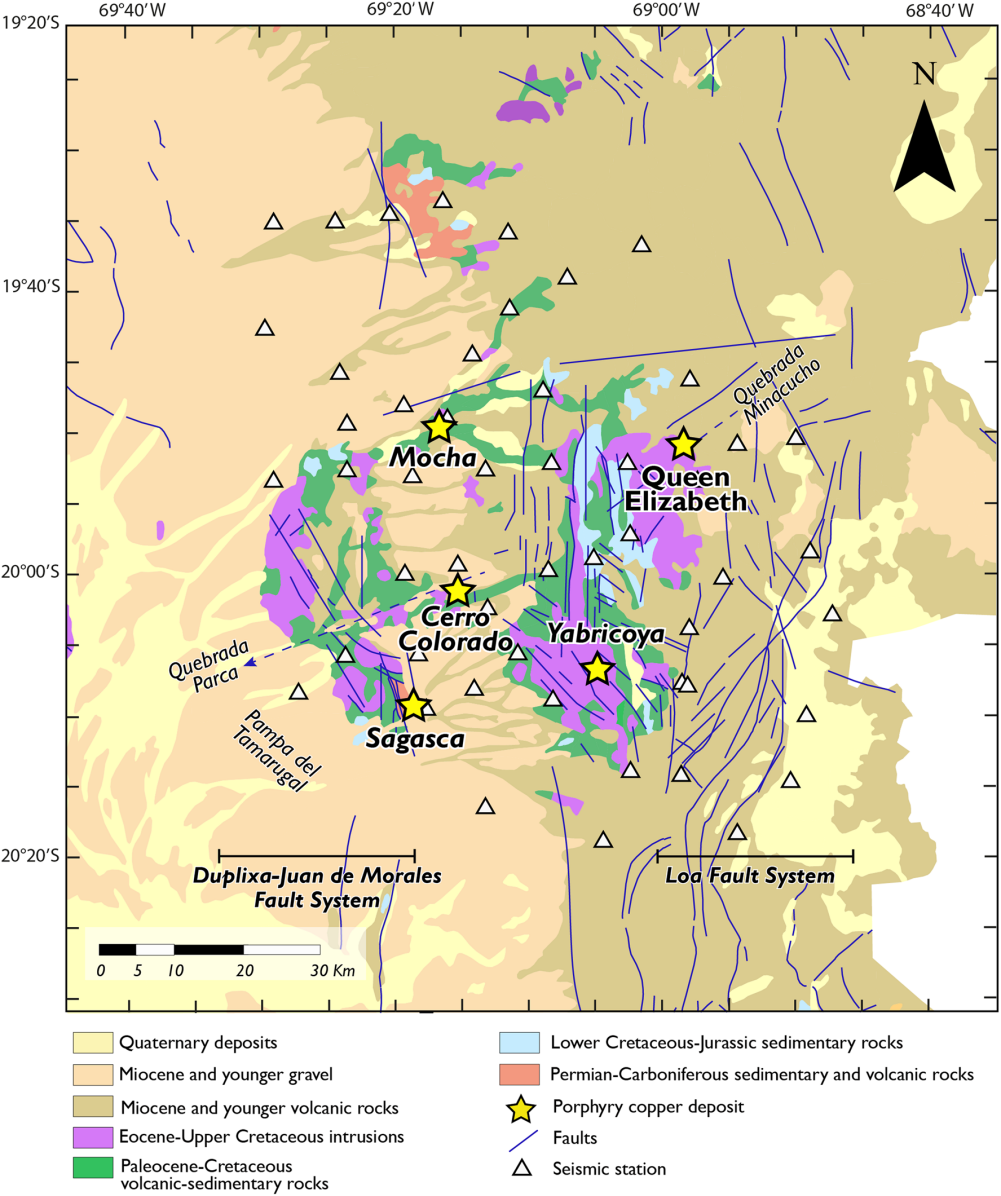
Geologic map of the study area (area shown in the rectangle in Fig. 1) with known porphyry copper deposits and prospects and main faults systems. Modified from Bouzari and Clark^26,27^ Morandé, et al.^31^ and Valenzuela, et al.^24^ The software used to generate this figure was Adobe Illustrator 2022 (www.adobe.com).

Our study area covers ~ 15,000 km^2^ between 19.2°S and 20.3°S in the Province of Tarapacá, northern Chile, and runs along the two main porphyry copper belts (Fig. 1). Cerro Colorado is the largest and only porphyry Cu–(Mo) deposit with an active mine site in the selected area, which includes several smaller porphyry prospects such as Mocha, and Sagasca along the Paleocene-Eocene copper belt, as well as Queen Elizabeth and Yabricoya to the east, along the Eocene–Oligocene copper belt. The area is dominated by gravel deposits of Miocene and younger ages to the West, covering the subplanar Pampa del Tamarugal, and by volcanic rocks of Miocene and younger ages to the east along the Western Cordillera (Fig. 2). The main structures in the area are northwest-trending faults, which depart from the generally north–south oriented faults associated with the Domeyko Fault System in the vicinity of Yabricoya and extend to the west and southwest of Cerro Colorado (Fig. 2). These northwest-trending faults partially delimit horsts of pre-Andean Upper Paleozoic basement and Mesozoic volcanic and sedimentary units which outcrop among the younger gravel deposits to the west of Cerro Colorado^25^. Uplift along these northwest-trending faults is believed to have started during the Late Eocene Incaic orogeny and is regarded as the main factor controlling the exposure and weathering of Paleocene to Lower Eocene porphyry copper deposits in the area^26,27^. Any porphyry center that would be located to the north or south of these uplifted blocks would be buried under thick gravel formations. Mesozoic volcanics and sediments underlying the Pampa gravels are also exposed along west-southwest-trending valleys such as the 300 m deep canyon of the Quebrada Parca*,* which runs along the northern rim of Cerro Colorado.

While the geological framework of the Cerro Colorado deposit has been well established by Bouzari and Clark^26,27^, the other copper prospects included in our study area have been poorly investigated. The Cu mineralization at Cerro Colorado is hosted by the Cerro Empexa Formation, a thick succession of mostly Upper Cretaceous andesitic volcanics and volcano-sedimentary rocks^26–29^. In the vicinity of the mine, the Cretaceous volcanics that unconformably overlie Late Paleozoic-Triassic basement granite are interpreted as the northernmost extension of the Choiyoi Magmatic Province^27–30^. The mineralization at Cerro Colorado is associated with porphyritic hypabyssal stocks which intruded the Cretaceous volcanics between 53–50 Ma, and with an earlier phase of magmatic activity at ca. 60–57 Ma^26–28^. A porphyritic intrusion of similar Upper Paleocene age (59–57 Ma) is found in the Mocha prospect, about 30 km to the North of Cerro Colorado^31,32^.

The north–south trending Domeyko Fault System is exposed along the eastern part of our study area. This orogen-parallel, 40–60 km wide zone of deformation stretches for more than a 1000 km along the Precordillera of northern Chile, and consists of an array of strike-slip, normal, and reverse faults, associated with folds and thrusts^21,33^. While the exact origin and deformation history of the Domeyko fault system are debated, a major tectonic pulse took place in the Middle Eocene to Early Oligocene, coinciding with the Incaic tectonic event, and the formation and emplacement of Eocene–Oligocene porphyry copper deposits in northern Chile^21,22^. In our study area, two main branches of the Domeyko Fault System striking roughly northward enclose the ~ 38 Ma Queen Elizabeth prospect^34^ exposed along the Quebrada Minacucho about 30 km to the northeast of Cerro Colorado. However, in contrast to other segments of the Domeyko Fault System where large porphyry deposits are found as discrete clusters in areas of long-lived focused magmatism^21^, Eocene–Oligocene porphyry copper prospects in our study area are low-grade and relatively small. This is the case of the Queen Elizabeth prospect, which shows minor supergene Cu enrichment that could be explained by high exhumation rates in the area and rapid burial by Late Oligocene–Miocene volcanic units^34^. The Eocene–Oligocene Yabricoya pluton and prospect to the southwest of Queen Elizabeth might also have been rapidly buried under a thick volcanic cover, hindering high supergene Cu enrichment in this area^34^.

### Hydrothermal alteration and mineralization

The Cerro Colorado Cu(Mo) porphyry deposit apparently is unique among documented central Andean porphyry systems in the association of sulfide mineralization with an intermediate argillic (quartz, sericite, and clay) to advanced argillic (andalusite, pyrophyllite, and diaspore) alteration^26,27^. These alterations are superimposed on earlier potassic-sodic alteration (biotite, albite, and magnetite) and transitional sericite-chlorite-clay alteration. The orebodies are dominated by quartz-sulfide stockworks of chalcopyrite and minor pyrite. In addition, molybdenite-bearing breccias occurred related to phyllic alteration (quartz, sericite, pyrite ± tourmaline)^26^. Mineralization at the Mocha deposit is characterized by quartz-chalcopyrite stockworks with minor supergene copper mineralization (chalcocite, malachite, and chrysocolla) associated with phyllic (quartz-sericite) alteration^32^.

## Methods and data processing

**Figure 3:**
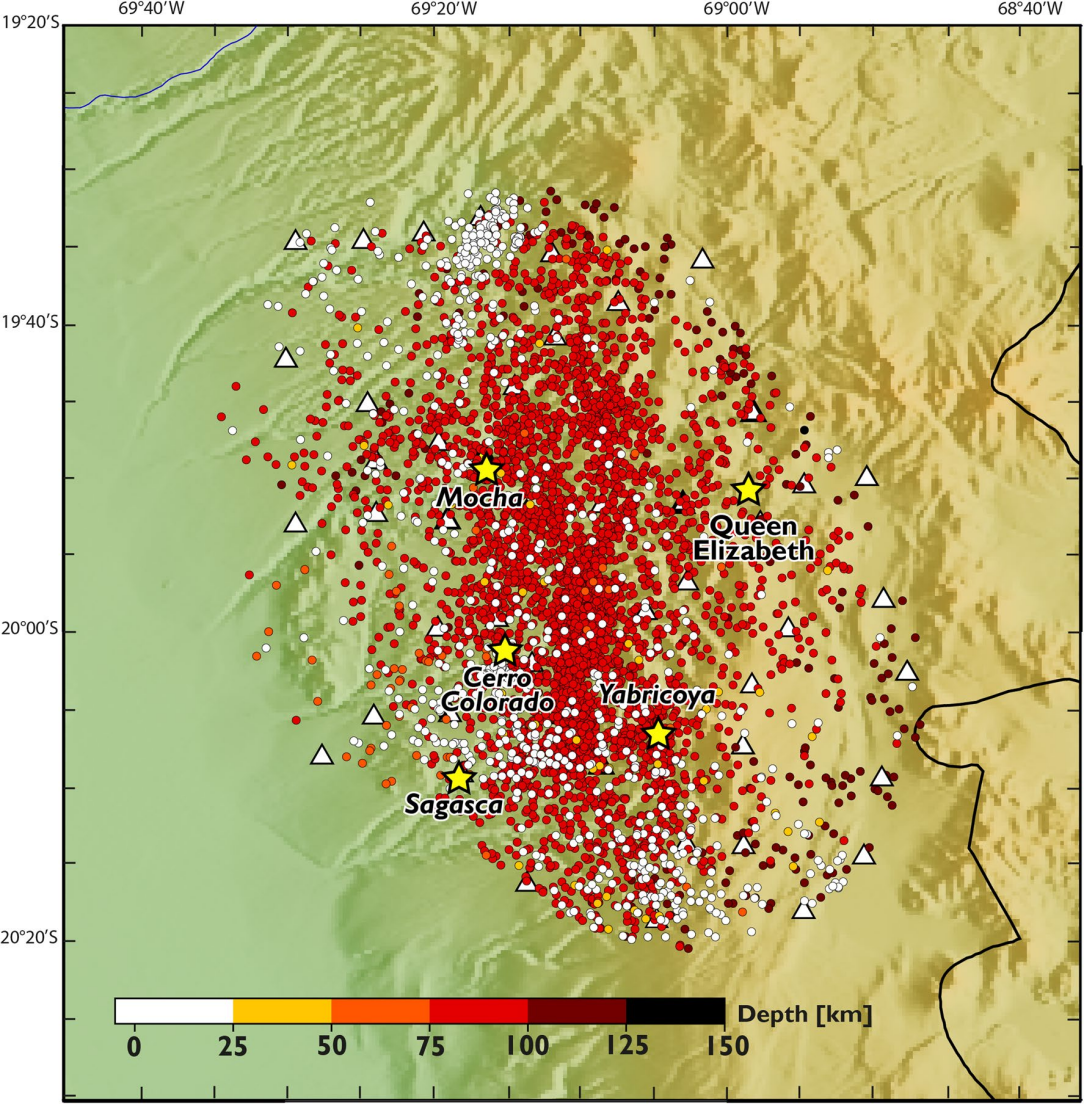
Distribution of the seismicity used for the Vp/Vs models determination. The color of the circles is associated to the depth scale shown in the bottom of the figure. White triangles correspond to the seismic stations, and yellow starts correspond to known porphyry copper deposits and prospects in the study area. The software used to generate this figure was QGIS 3.22 (www.qgis.org).

The data included in this work correspond to the P- and S- wave arrival times generated by seismicity recorded by the seismological stations deployed in the study area between October 9, 2018, and June 28, 2019 (Fig. 3). The temporary seismic network was composed by 51 short period (4.5 Hz), 3 components, continuous recording stations. The P- and S-wave arrival times were estimated using the Regressive Estimator (REST) autopicking package 16. REST generates hypocenter catalogs by combining the autoregressive approach of Pisarenko et al.^35^ and Kushnir et al.^36^ with windowing algorithms of Rawles and Thurber^37^. As part of this procedure, we parameterize the subsurface by a three-dimensional grid of nodes that covers a volume of 31 × 32 × 38 km^3^ (west–east, north–south, and depth, respectively) at an evenly spaced interval of 4 km, for a total of 37,696 nodes. The 38 elements along vertical axis cover a total of 145 km, from 5 km above sea level to a depth of 140 km. Initial event locations were determined by REST using a 1D velocity model following the procedure of Comte, et al.^14^ The recorded seismicity generated 103,247 P and 101,696 S arrival times, or 204,943 total observations that were used for wave speed inversion.

The initial velocity model corresponded to the same 1D model used in the autopicking and an initial ratio Vp/Vs = 1.82 that was determined (Appendix 1; Fig. S1). As our focus is on Vp/Vs ratios, we jointly solve for Vp and Vp/Vs and recover Vs by division. The inverse problem involves the simultaneous determination of hypocenters and wave speed perturbations using damped least squares. Regularization was achieved by adding a standard damper, and by twice applying a moving average window over the perturbations determined by the inversion that averages 5 values on each of all directions (latitude, longitude, depth). As stable hypocenters generally promote robust convergence, we required: (i) that all events be associated with a minimum of 8 phases; (ii) that any phase has an absolute travel time residual less than 1.5 s; (iii) that the standard deviation for all the residuals for an event has a maximum of 2 s; and (iv) that the azimuthal gap of stations recording the event be less than 220° generating a final catalog of 10,223 earthquakes for the inversion.

To evaluate the resolution capabilities of our data set, we ran standard checkerboard tests for both Vp and Vs (Appendix 2). We perturbed the initial 1D models with anomalies of ± 5% in square prisms of 10 × 10 km^2^ dimension in latitude and longitude, and variable length in depth. The first anomaly is 10 km deep, the second and the third anomalies are 20 km deep, and the fourth anomaly is 30 km deep (Fig. S3). Based on the results of this checkerboard test shown in plain view (Figs. S4 and S5) and in-depth sections (Figs. S6 and S7), we estimate that we have good resolution at a scale length of 10 km under the porphyry system studied here, from the surface down to 90 km depth.

### Vp/Vs ratio

In this study we focus on the ratio of compressional (P) and shear (S) wave velocities, i.e., Vp/Vs, to determine new constraints on deep seated geologic structures that shed light on the architecture of subsurface porphyry copper deposits. We note that variations in Vp/Vs reveal patterns of anomalies because Vp/Vs ratio is inversely proportional to rigidity of the rocks.

The Vp/Vs ratio has long been shown to vary as a function of the nature and composition of rocks^37^. Seismic velocities are also sensitive to physical parameters such as pressure and temperature conditions, porosity and pore geometry, fracture density, and the presence of fluids^39–41^. The influence of all these factors on seismic velocities means they are often difficult to interpret uniquely. At the same time, Vp/Vs ratios have been shown to reduce this ambiguity. For example, in volcanic systems, while Vp has been shown to be primarily sensitive to rock composition and Vs to the presence of fluids e.g., Koulakov et al.^12^, the Vp/Vs ratio can be used to distinguish hydrothermal fluids from partial melts as well as gas-bearing from liquid-bearing rocks^11^.

We note that despite its demonstrated efficacy in subsurface imaging, local earthquake tomography has only recently been applied to brownfield and greenfield mineral exploration worldwide, and much remains to be learned about the significance of Vp/Vs anomalies in ore systems.

### Seismic tomography results

**Figure 4:**
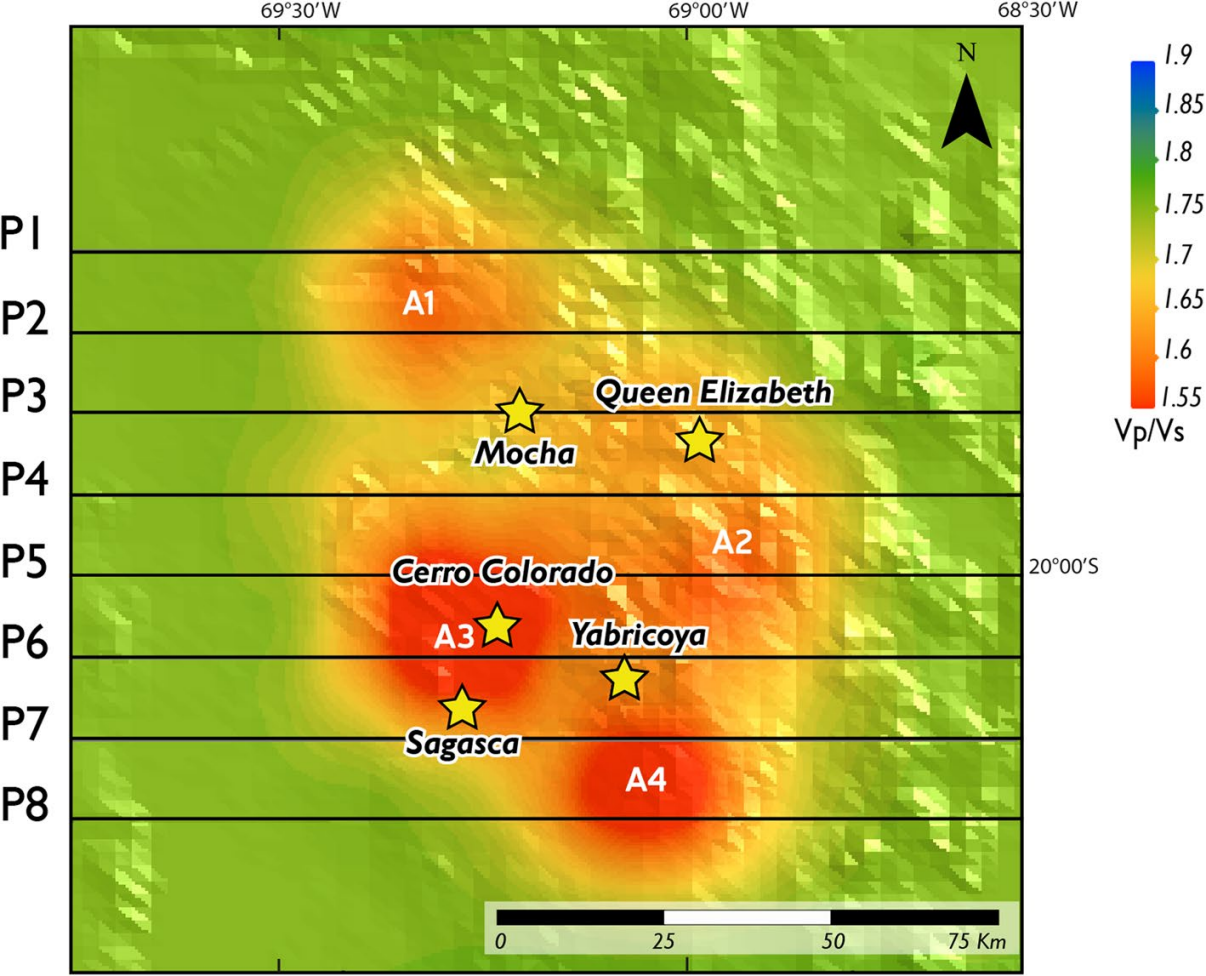
Plan-view map for Vp/Vs ratios of the study area (Figs. 2 and 3). The black lines denote the extracted profiles for the vertical Vp/Vs vertical sections shown in Fig. 5. The yellow stars correspond to historical porphyry copper deposits and prospects included in the study area. A1 to A4 correspond to the *low Vp/Vs* anomalies described in the text. Data georeferencing was carried out with ArcGIS Pro10.1 (www.esri.com). The software used to generate this figure was Leapfrog 2022.1 (www.seequent.com) and the final editing was done with Adobe Illustrator 2022 (www.adobe.com).

**Figure 5:**
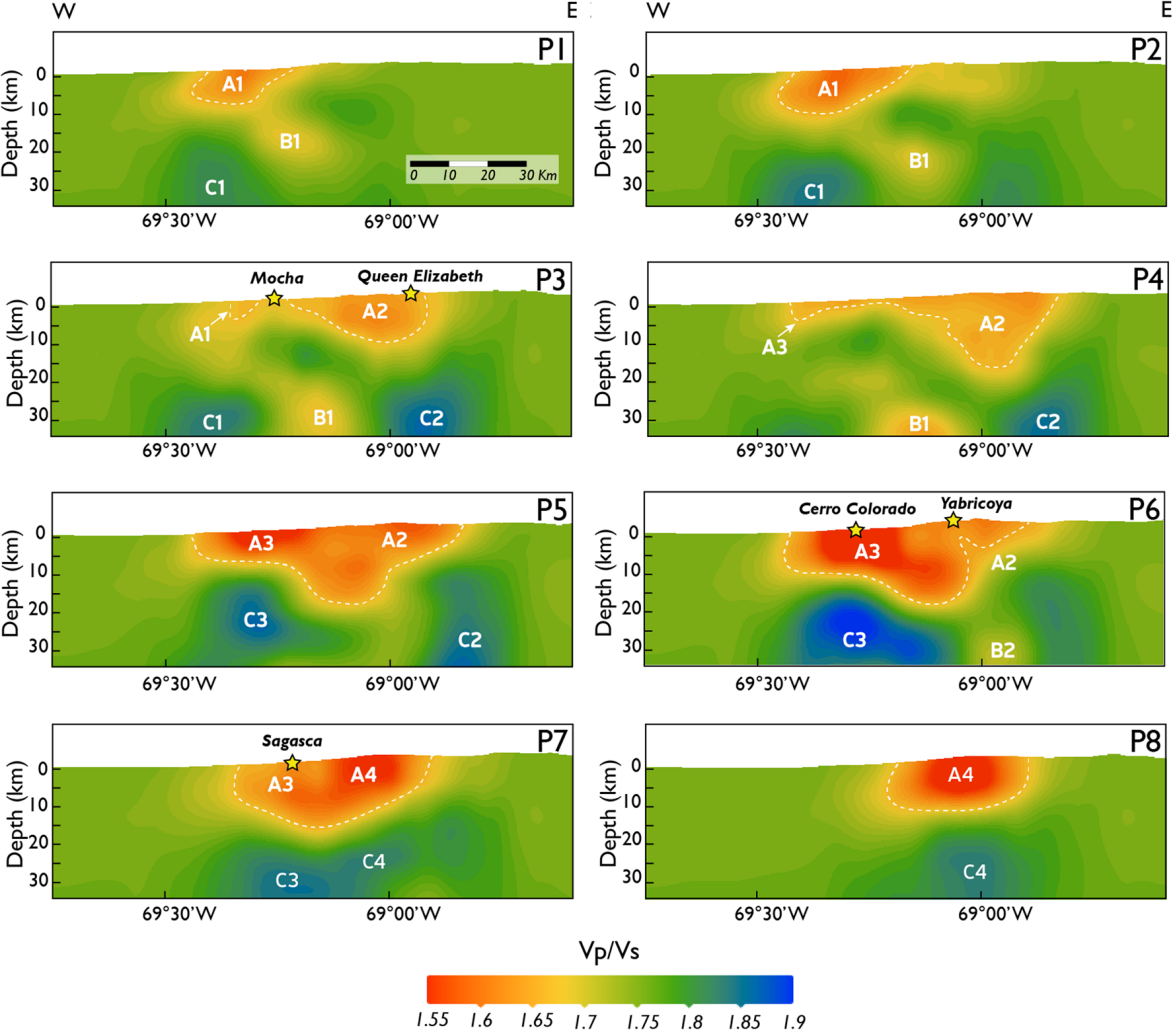
Vertical sections for Vp/Vs ratios along profiles P1-P8 (see Fig. 4 for map location). The white dotted curves denote *low Vp/Vs* areas. The yellow stars (surface) correspond to historical porphyry copper deposits and prospects included in the study area. A (A1 to A4), B (B1 and B2) and C (C1 to C4) anomalies correspond to the *low, medium,* and *high Vp/Vs* anomalies, respectively, described in the text. Data georeferencing was carried out with ArcGIS Pro10.1 (www.esri.com). The software used to generate this figure was Leapfrog 2022.1 (www.seequent.com) and the final editing was done with Adobe Illustrator 2022 (www.adobe.com).

The local earthquake tomography results are shown in Figs. 4 and 5 including the Vp/Vs ratios in map view and vertical sections, respectively (Vp and Vs are shown in Appendix 3; Figs. S7 and S8). It is important to point out that along the study area, we have a sufficient in-depth and spatial resolution, according to the checkerboard tests' results (Appendix 2). To facilitate the discussion, we characterize values of Vp/Vs ratios as: *low Vp/Vs* (~ 1.55–1.65), *medium Vp/Vs* (~ 1.65–1.75), *high Vp/Vs* (~ 1.75–1.85), and *very high Vp/Vs* (~ 1.85–1.90).

The map view and vertical sections show four prominent *low Vp/Vs* anomalies, (A1 to A4 in Figs. 4 and 5). These *low Vp/Vs* anomalies match the locations of known porphyry copper deposit and prospects within the study area and extend to ~ 5–15 km depth (Fig. 5). The first (A1) and the second (A2) Vp/Vs anomalies are located northwest of the Mocha prospect and southeast of Queen Elizabeth prospect, respectively. The third Vp/Vs anomaly (A3) coincides with the location of the Cerro Colorado deposit and is bounded to the south by the Sagasca prospect. The fourth Vp/Vs anomaly (A4) is located immediately south of the Yabricoya prospect (Fig. 5). *Medium Vp/Vs* anomalies (B1 and B2) are found below the A1 and A2 anomalies in the deep part of vertical sections P1 to P4 (~ 10–35 km) (Fig. 5). In vertical section P3, the B1 anomaly appears to extend to a shallower level in a channel like-structure (Fig. 5). *High Vp/Vs* anomalies (C1 to C4) are found in vertical sections P1 to P8, at depths greater than 15 km (Fig. 5). The most prominent *high Vp/Vs* anomaly, the C3 anomaly, is located just below the Cerro Colorado deposit (vertical section P6; Fig. 5).

The three-dimensional (3D) local earthquake tomography model is shown in Appendix 4, including the Vp/Vs ratios in perspective and plan views (Fig. S9). This model shows highly heterogeneous structures corresponding to connected and isolated bodies characterized by *low Vp/Vs*, *medium Vp/Vs*, and *high Vp/Vs* anomalies (Fig. S9). Notably, these bodies correlate with the A, B and C anomalies identified in vertical sections (Fig. S9a). Below 35 km depth, *low* and *medium Vp/Vs* anomalies appear as concentric bodies composed of an inner core (A anomalies), surrounded by an outermost shell (B anomalies) (Figs. S9b,c). Moreover, the study area is divided into northern and southern domains. The northern domain includes the A1 anomaly alone, whereas the southern domain includes the A2, A3 and A4 anomalies which are connected to each other in depth (Fig. S9c). Similarly, the SSE-NNW oriented channel-like structure, previously described in vertical section P3 (Fig. 5), separates the system into these two domains, and connects the deeper B1 body with the shallower A1 anomaly (Fig. S9c). Prominent volumes of connected and isolated bodies characterized by *high Vp/Vs* to *very high Vp/Vs* ratios are observed at depths greater than 35 km, and a small drop-shaped body (B3) of *medium Vp/Vs* ratio is seen in the lower part of the model (~ 100 km) (Fig. S9a).

## Discussion

### Significance of Vp/Vs anomalies

Based on the vertical sections through the 3D model, we identify bodies characterized by *low Vp/Vs* (A bodies), *medium Vp/Vs* (B bodies), and *high Vp/Vs* (C bodies) anomalies (Figs. 5 and S9), that we attribute to the presence of magmatic reservoirs, and fluid and melt zones beneath the study area.

At depths shallower than 15 km, *low Vp/Vs* anomalies (A1 to A4 bodies) correlate with the location of porphyry copper deposits and prospects included in our study area, i.e., Cerro Colorado, Mocha, Queen Elizabeth, Yabricoya, and Sagasca (Figs. 5 and S9). The reduction of the Vp/Vs in the subsurface is characteristic of various types of hydrothermal ore deposits including IOCG^17^, porphyry and epithermal Au-(Cu)^20^, and porphyry Cu-(Mo) deposits^16,18^. Based on previous studies, we infer that *low Vp/Vs* anomalies in the study area are probably be due to a higher concentration of low-elastic minerals, e.g., quartz with impregnated sulfides, compared to the mineral assemblages that compose the host rock^42^; the occurrence of hydrothermally altered zones^43–45^; and, the presence of clay-filled fracture zones, as well as water-saturated fractures that can cause anomalously low Vp and Vs^46,47^.

Recently, Spichak and Goidina^18^ built a 3D model that shows the strong correlation between *low Vp/Vs*, low electrical resistivity and low-density domains beneath the Sorskoe Cu-Mo deposit in Russia down to a depth of 45 km. These geophysical signatures were interpreted to indicate the presence of aqueous fluids in fractured zones, stockworks impregnated with sulfides, as well as thick metallic veins. Accordingly, at Cerro Colorado, the largest porphyry copper deposit in our study area, the occurrence of highly fragmented structures, including breccias, veins and stockworks filled with sulfides, quartz, and clays^26^, could explain the *low Vp/Vs* anomaly associated with the deposit. By analogy, we suggest that in our study area the orebodies and associated hydrothermal alteration are confined within *low Vp/Vs* domains.

At depths greater than 20 km, we interpret *medium Vp/Vs* (B bodies) and *high Vp/Vs* (C bodies) anomalies as magma reservoirs, intrusive plutons and melt conducts. On one hand, *medium Vp/Vs anomalies* (B bodies) occur just below and around *low Vp/Vs* anomalies (A bodies) and are also characterized by higher percentage variation of Vp than the surrounding media (Figs. S7 and S9c). We therefore infer that B bodies correspond to intermediate to felsic intrusions related to porphyry copper deposits that have probably cooled down. This idea is supported by the fact that felsic bulk composition (e.g., granodiorite) leads to Vp/Vs ~ 1.7^38^, as well as the presence of outcropping intermediate to felsic intrusions in the study area (Fig. 2). It follows that the channel-like feature (B1 body), characterized by *medium Vp/Vs* ratios could correspond to a melt conduit (Fig. S9c). Since we do not observe a strong attenuation in the percentage variation of Vs (Fig. S2), we suggest that no partial melt is left. Moreover, we propose that the intersection of different fault planes, related to the Duplixa–Juan de Morales and Loa fault systems (Fig. 2), may produce this conduits for the migration of magmatic and hydrothermal fluids^48^.

Additionally, C bodies are characterized by the absence of seismicity, *high Vp/Vs* anomalies (Fig. S9a), and areas with mostly low percentage variations of Vp and Vs (Figs. S7 and S8). We therefore interpret *C* bodies as mafic magmatic reservoirs. This is consistent with experimental studies that suggest that Vp/Vs increases are associated with high temperatures and melts, compared to Vp/Vs decreases that may be related to high gas content or supercritical fluids^49^. Consistently, mafic bulk composition of rocks leads to high Vp/Vs ~ 1.85 (e.g., gabbro)^38^. Finally, the deep drop-shape body characterized by *medium Vp/Vs* (B3 body; Fig. S9a) may not represent a felsic composition. Rather, the difference between B3 and C bodies could be due the presence of partial melt in C, reducing Vs and therefore increasing Vp/Vs.

### Crustal architecture and its relation to porphyry mineralization

The spatial distribution of ore deposits is mainly influenced by crustal architecture, which is partly controlled by tectonic and geodynamic processes, due its impacts on the flow of fluids throughout the crust^50^. Thus, crustal features and structural framework inferred from local earthquake tomography can lead to a better understanding of the development of copper metallogenic belts in northern Chile. In porphyry copper systems, crustal thickness is considered a first-order control on the duration and volume of magmatic activity, metal and fluid sources, ore-forming processes, fluid pathways, ore deposition sites, and the emplacement depth of orebodies^7^.

**Figure 6:**
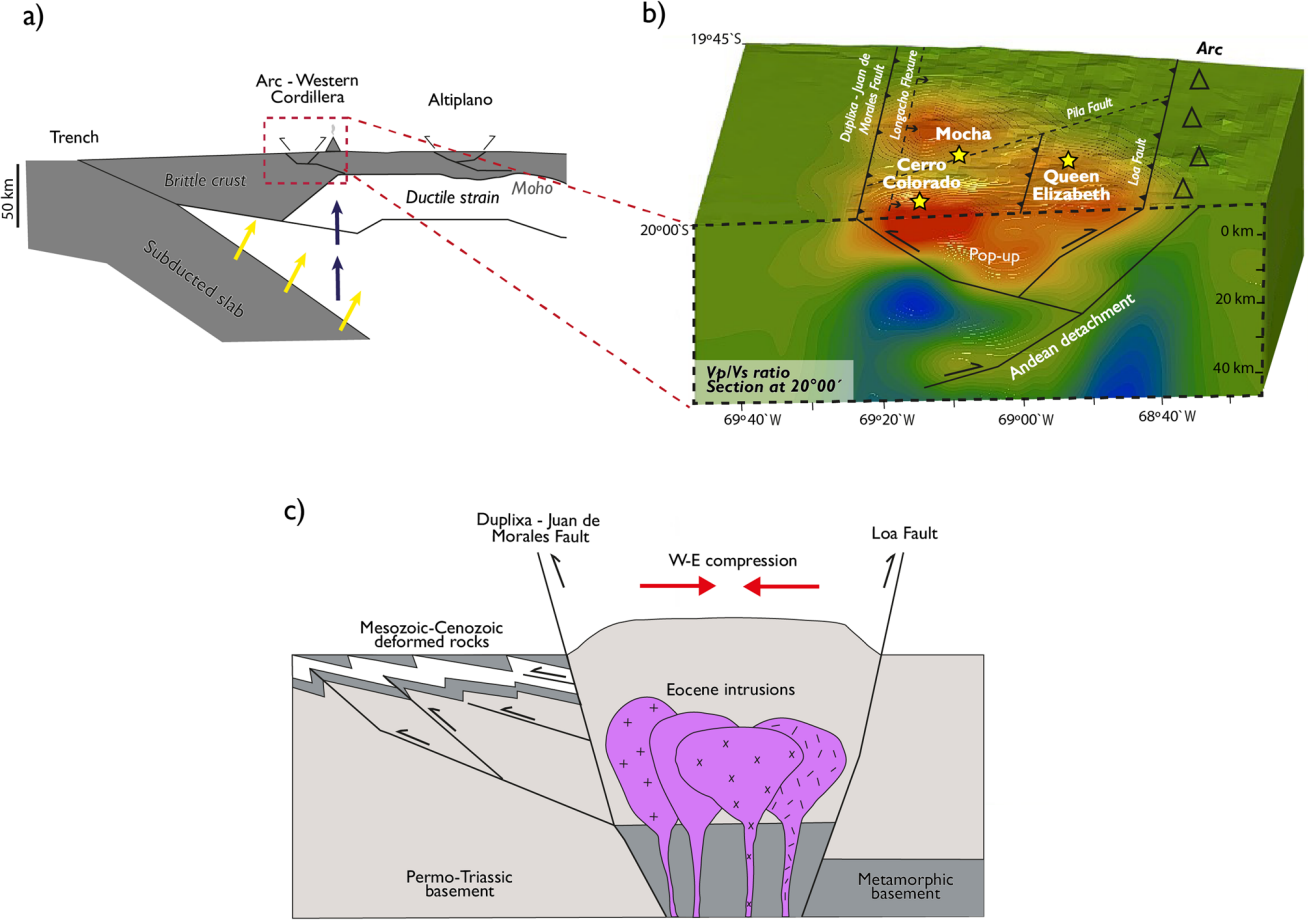
Subsurface architecture beneath the Central Andes subduction zone and the study area. (**a**) Schematic representation of the Central Andean subduction zone based on seismic and magnetotelluric models^13,15^. The subduction slab dehydration (yellow arrows) causes mantle wedge serpentinization and provides the flux needed to lower the melting temperature. At depths of 120–150 km, partial melting of ultramafic mantle rocks occurs yielding to the generation of basaltic magmas (blue arrows). The figure also shows the location of the Western Cordillera magmatic arc. (**b**) Proposed subsurface architecture in the study area based on Vp/Vs model. Figure shows inferred pop-up structures rooted in the Andean east-verging detachment within a regional context dominated by compression^55^. (**c**) Eocene porphyry intrusion emplacement model in a pop-up geometry. Figure (**a**) was generated by the software Adobe Illustrator 2022 (www.adobe.com) and based on Comte et al. ^13^. Figure (**b**) was generated by Leapfrog 2022.1 (www.seequent.com) and the final editing was done with Adobe Illustrator 2022 (www.adobe.com). Faults distribution are based on Valenzuela, et al.^24^. Figure (**c**) was generated by software Adobe Illustrator 2022 (www.adobe.com) and modified from Masterman, et al.^59^.

The crust and upper mantle architecture beneath the Nazca-South America Plate boundary between ~ 18ºS and 24°S in northern Chile have been previously revealed using local earthquake tomography^13^ (Fig. 6a). Beneath the volcanic arc, a total continental crust thickness of ~ 50–65 km has been determined^13,15^. The upper continental mantle is characterized by Vp/Vs ~ 1.76, whereas the lithospheric mantle wedge is characterized by Vp/Vs ~ 1.76–1.8 related to the mantle serpentinization^13,38^. The location of continental Moho has been estimated at ~ 50 km depth using P-to-S converted teleseismic waves in northern Chile^51^, ∼35 km depth using a morphometric analysis combined with a numerical model of landscape evolution to estimate uplift rates along the central Andean^52^, and ∼40 km depth presenting a density-depth model along the Nazca-South America subduction margin, from 18°S to 23,5°S^53^. The lower portion of the continental crust is composed of crystallized melts that underplate the crust causing its thickening continuously as the magmatic arc migrated progressively eastward^15^. Eastward migration of the magmatic arc in northern Chile started at least during the Jurassic causing the migration of the volcanic arc from the Coastal to Western Cordillera^53^.

From the structural standpoint, seismic tomography models in this study are consistent with the regional geology (Fig. 2). *Low Vp/Vs* anomalies coincide with the occurrence of pop-up structures and typically positive flower structures along two clear principal faults, including the Duplixa-Juan de Morales fault in the west, and the Loa fault in the east (Fig. 6b). Pop-up structures have been previously described as part of the Andean architecture, including the Miocene West vergent thrust system that dominates the north of Chile between 18°S and 21°S^55,56^, and the Mesozoic basin structures reactivated in the Late Cretaceous and Cenozoic^57^*.* Figure 6b shows the latitudinal extension of this bivergent structure, being the convergence of the eastern and western limits around ~ 20–25 km depth, which is consistent with the plausible position of the Andean detachment in the area^55,58^. We interpret that the emplacement of porphyry intrusions in the crust is favored by these pop-up geometries, as previously proposed for the Collahuasi Cu-(Mo) porphyry deposit^59^ that is located in the Late Eocene-Early Oligocene copper belt further south of the study area (Fig. 1). Our seismic tomography results show that crustal architecture—including major crustal structures—acts as a first-order control on the location of copper metallogenic belts in northern Chile.

### Three-dimensional conceptual model

**Figure 7:**
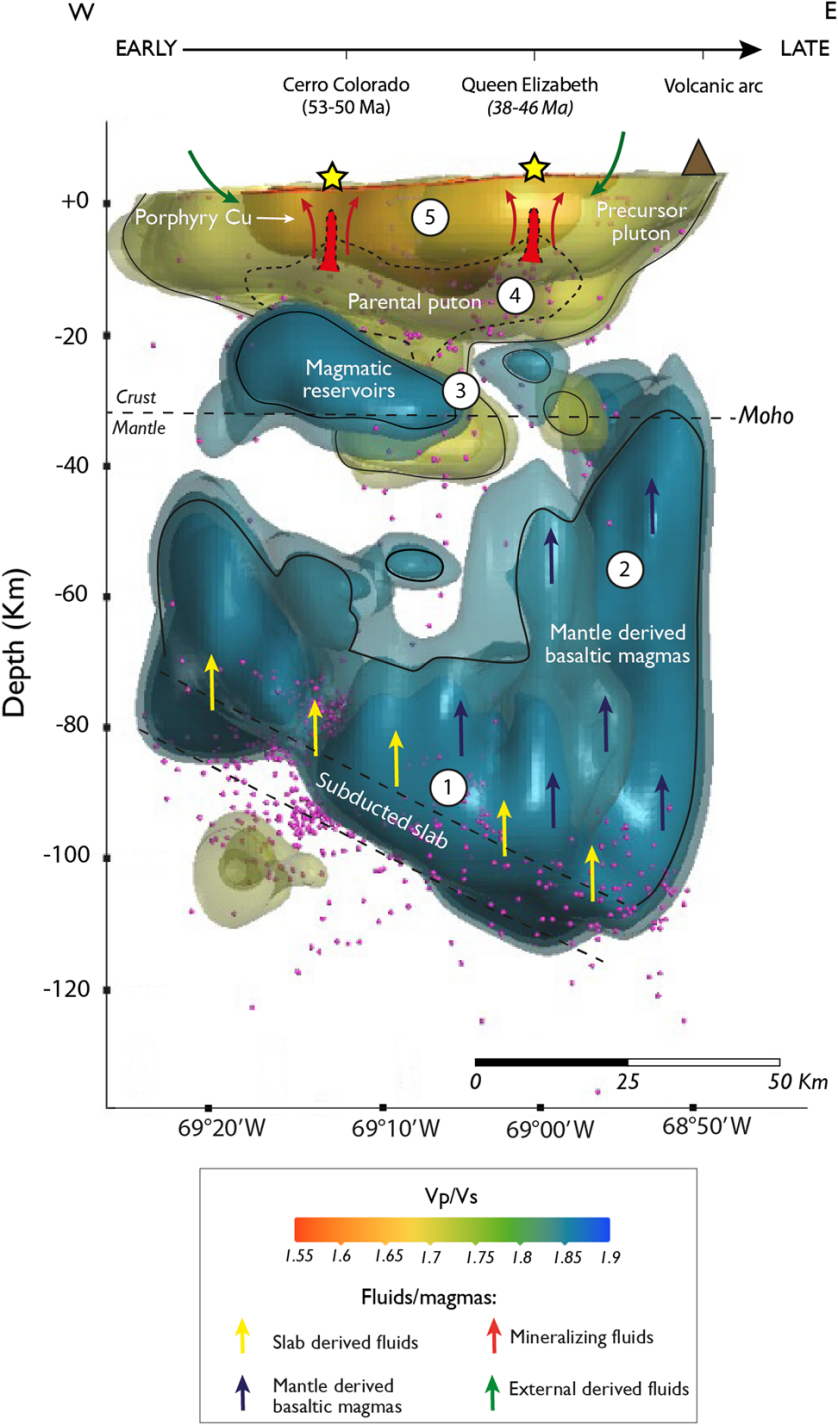
Conceptual model for the formation of the porphyry copper system beneath the study area based on the distribution of the Vp/Vs ratios (see also Supplementary Video S1). Arrows indicate the possible paths of fluids and/or magmas. The dotted lines indicated inferred features and pink circles correspond to the seismicity recorded in the area. We used an average Moho depth from Melnick^52^ and Maksymowicz, et al.^53^ The numbers indicated inside the circles correspond to the stages of the porphyry system formation *(Stages 1 to 5*) explained in the main text. See text for discussion. Data georeferencing was carried out with ArcGIS Pro10.1 (www.esri.com). The software used to generate this figure was Leapfrog 2022.1 (www.seequent.com) and the final editing was done with Adobe Illustrator 2022 (www.adobe.com).

A conceptual model shows the subsurface architecture down to a depth of 120 km, denoting the inferred magmatic reservoirs, intrusive bodies, and fluid pathways that formed the porphyry copper system beneath the study area (Fig. 7; Video S1). We coupled 3D visualization of the Vp/Vs model (Fig. S9) with widely accepted formation models for porphyry copper deposits^3,7,8^. Our conceptual representation is consistent with the subduction architecture of the Andean margin (Fig. 6a) and the migration to the east of the magmatic arc during Cenozoic times. The latter is supported by U–Pb zircon geochronology of intrusive porphyries and the superficial expression of porphyry copper deposits, in which the younger Queen Elizabeth prospect (~ 46–38 Ma^34^) is located further east with respect to the older Cerro Colorado deposit (~ 53–50 Ma^29^). Furthermore, the relative position of the modern volcanic arc is revealed by low Vp and low Vs anomalies in the easternmost part of the study area, that we interpret as melt migration (Figs. S7 and S8). Consistently, this location correlates with that of the active Isluga volcano that is located further north of the study area at longitude 68.83ºW.

Our proposed conceptual model in Fig. 7 considers a series of stages to explain the formation of the porphyry system beneath the study area. The presence of the subducted slab dipping toward the east, at more than 100 km depth, is inferred by the intense seismic activity, as well as by the *very-high Vp/Vs* ratio of the bodies in the deeper portion of the model (Fig. S9a). Note in this relation that Vp/Vs ratios ~ 1.9–2.0 are consistent with hydrated mantle rocks such as serpentinite^13,38^. *Stage 1* comprises the release of oxidizing fluids from the subducting slab causes the hydration of the mantle wedge, followed by partial melting of the mantle that produces oxidized, hydrous basaltic arc magmas^7^. During *stage 2,* the multiple pulses of basaltic arc magmas, represented by rising bodies characterized by *high Vp/Vs* ratios, ascend and later pool at the mantle-crust boundary (i.e., Moho), forming multi-depth reservoirs in the mid to lower crust (~ 30–70 km depth)^7^. *Stage 3* involves differentiation of basaltic magmas by fractional crystallization, crustal assimilation, recharge, and mixing processes^7^. Importantly, the depth of magmatic differentiation is fundamentally controlled by the crustal thickness and tectonic environment^60^. These magmatic reservoirs could be treated as the potential source for Cu-rich mineralizing fluids, which are involved in hydrothermal processes in the lower-middle crust at the stage of development of ore-bearing porphyry intrusions. During *stage 4*, the evolved magmas ascend to form upper crustal magma chambers at depths of ~ 5–15 km^8^, i.e., the parental plutons for porphyry copper formation. These magmatic reservoirs are periodically fed by magmas from the lower crustal reservoirs^3,7^. Decompression, degassing, metamorphism and differentiation processes result in the exsolution of ore-forming fluids that are capable of scavenge significant amounts of metals from magmas^3,7,60^. Porphyry Cu-related intrusions comprise composite precursor plutons, parental plutons, as well as multiple phases of the plug-like porphyry copper intrusions that were emplaced at depth of 1–7 km^3,7^. We interpret that the underlying intrusive suite of rocks characterized by *medium Vp/Vs* ratios act as hosts for a cluster of copper deposits in the study area (see Cerro Colorado and Queen Elizabeth in Fig. 7). The final stage (*stage 5*) comprises the formation of porphyry copper mineralization that occur around plug-like intrusions by the precipitation of Cu-sulfides from ore-forming fluids that are released from these plugs^3,7,8^. In the upper crust, where plastic deformation changes to brittle, the transport of fluids mainly occurs along a network of pores and fractures^18^. Therefore, multiple events of hydrothermal alteration and mineralization occur as stockworks and breccias, that are typical structures of systems dominated by high water/rock ratios^3^. *Stage 5* is accompanied by *low Vp/Vs* anomalies along fluid pathways conduicts, hydrothermally altered zones, and metallic ore deposition and accumulation sites^18^.

### Implications for exploration of porphyry Cu deposits

Globally, the exploration and exploitation of porphyry copper resources is moving to greater depths^1^. Exploration efforts are influenced by the fact that most of the shallower and outcropping deposits have been discovered in northern Chile. Moreover, exploration has been historically focused on brownfield environment, where the inherent clustering characteristics of porphyry copper formation makes this a conceptually lower risk investment. Currently, opportunities towards new discoveries of world-class deposits are focused on greenfield exploration, especially under post-mineralization cover or blind targets along the copper metallogenic belts in northern Chile.

The challenge of discovering new porphyry copper deposits requires an improvement of the lateral and vertical resolution of deep-seated orebodies for targeted mining. We demonstrate the potential of local earthquake tomography to delineate structures hosting porphyry copper systems. Our Vp/Vs models strongly supports the notion that the crustal architecture, e.g., pop-up geometries, acts as first-order control on the location of the orebodies (Fig. 7), which may also have implications for ore genesis and intrusion emplacement models. Moreover, the size of lower crustal reservoirs may be an important factor contributing to porphyry mineralization, allowing long-lived magmatic activity^7^. Interestingly, the largest high Vp/Vs (C anomaly) is below Cerro Colorado, the only large deposit in our study area (vertical section P6 in Figs. 5, S9a).

We emphasize that local earthquake seismic tomography by itself may not sufficiently discriminate potentially mineralized zones from related hydrothermally altered zones and the enclosing host rock. Future work will focus on integrated 3D models though multi-source data, for example by combining 3D geophysical modeling with geological, geochemical, and structural interpretation, remote sensing, and pattern recognition through machine learning. Ultimately, we recommend seismic tomography as a valuable and environmentally friendly tool to identify new porphyry copper deposits, and probably other types of magmatic-hydrothermal ore systems for instance iron oxide-copper gold (IOCG) deposits.

## Final remarks

We obtained high-resolution visualization of the deep-seated structures beneath a porphyry copper system in northern Chile using local earthquake arrival time tomography. The obtained 3D Vp/Vs model provide insights into porphyry copper deposits on a regional scale, from the perspective of crustal architecture, fluids/melt pathways and reservoirs, as well as mineralized and hydrothermally altered zones. Although seismic tomography still cannot directly image mineralization zones, it can provide good indicators of the structures that host porphyry copper deposits. In the drive to discover new mineral resources, local earthquake tomography could be a powerful and effective approach to look for concealed deposits with minimal impacts on the environment. Our results demonstrate that Vp/Vs models open new avenues to image the subsurface architecture of magmatic-related ore systems from the upper mantle to the surface, and therefore could be used for the exploration of deep and undercover orebodies.

## Supplementary Information

Supplementary Information 1.

Supplementary Video 1.

Supplementary Table S1.

## Data Availability

All data generated and analysed during this study are included in this published article in the Supplementary Material (Excel file; Table S1).
