# Supplementary material for: Imaging the subsurface architecture in porphyry copper deposits using local earthquake tomography
Source: Sci Rep. 2023 Apr 26;13:6812. doi: 10.1038/s41598-023-33820-w (PMC10133302; doi:10.1038/s41598-023-33820-w)
Supplement: Supplementary file 1 — Supplementary Information 1. [file 41598_2023_33820_MOESM1_ESM.pdf]

Supplementary Material

Imaging the subsurface architecture in porphyry copper deposits using local earthquake tomography

Diana Comte[1,2], Gisella Palma[2,3,*], Jimena Vargas[4], Daniela Calle[2], Matías Peña[2,3], Sergio García-Fierro[2,3], Joëlle D'Andres[2,5], Steven Roecker[6], Sergio Pichott[4]

[1] Departamento de Geofísica, Facultad Ciencias Físicas y Matemáticas, Universidad de Chile, Santiago 8370449, Chile
[2] Advanced Mining Technology Center, Universidad de Chile, Santiago 8370451, Chile
[3] Escuela de Geología, Universidad Mayor, Av. Manuel Montt 367, Santiago, Chile
[4] CODELCO, Gerencia de Exploraciones, Casa Matriz, Huérfanos 1270, Santiago, Chile
[5] Research School of Earth Sciences, the Australian National University, Canberra, ACT 2601, Australia
[6] Earth and Environmental Sciences, School of Science, Rensselaer Polytechnic Institute, Troy, NY 12180, USA

*****Corresponding author: gisella.palma@umayor.cl

JOURNAL NAME: Scientific Reports

Appendix 1. Methods and data processing

Table S1. P and S wave velocities (km/s) and Vp/Vs ratio used for this study (Excel file).

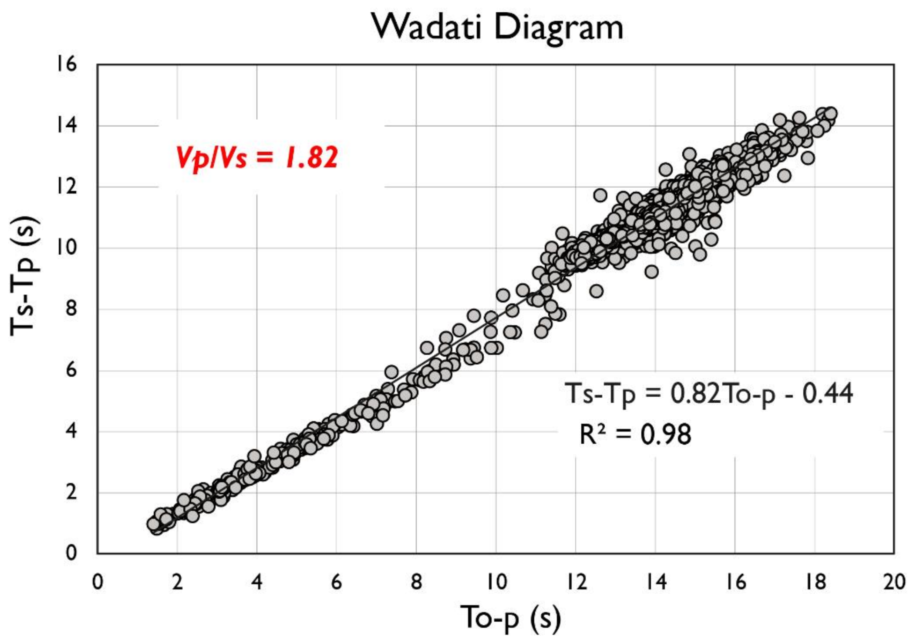

Figure S1. Wadati Diagram (Wadati, 1933) between difference in travel time Ts-Tp (y-axis) and To-p (x-axis). The slope of the best fit line was used to obtain the corresponding Vp/Vs ratio (Vp/Vs=1.82) of the origin time of local earthquakes. The software used to generate this figure was Microsoft Excel (www.microsoft.com), and the final editing was done with the software Adobe Illustrator 2022 (www.adobe.com).

Appendix 2. Checkerboard Test

To evaluate the resolution capabilities of our data set, we ran standard checkerboard test for both Vp and Vs. We perturbed the initial 1D models with anomalies of ± 5% in square prisms of 10 × 10 km^2 dimension in latitude and longitude, and variable length in depth. The first anomaly is 10 km deep, the second and third anomalies are 20 km deep, and the fourth anomaly is 30 km deep. These perturbations start from 0 km depth, and each anomaly is 5 km apart in all 3 directions (Fig. S2). The results in plan view (Figs. S3 and S4) suggest that resolution is acceptable until 70 km depth where main porphyry copper deposits are located (red stars in Figs. S2a, S3 and S4). Between 70 to 90 km the resolution decreases for the three northernmost deposits (i.e., Mocha, Queen Elizabeth, and Cerro Colorado), and is lost for the southernmost deposits (i.e., Sagasca and Yabricoya). Figures S5 and S6 show good resolution for small anomalies in shallow (i.e., first 10 km depth). Between 10 and 60 km depth, resolution is good for anomalies that extend over 20 km in depth. Between 60 to 90 depths, resolution is good for the large anomalies of the three northernmost deposits but is lost for the southernmost deposits (i.e., the same pattern as seen in plan view).

Based on the checkerboard test results, we consider that the resolution to study porphyry copper deposits in the study area at the scale of 10 km laterally and 10-20 km vertically is acceptable.

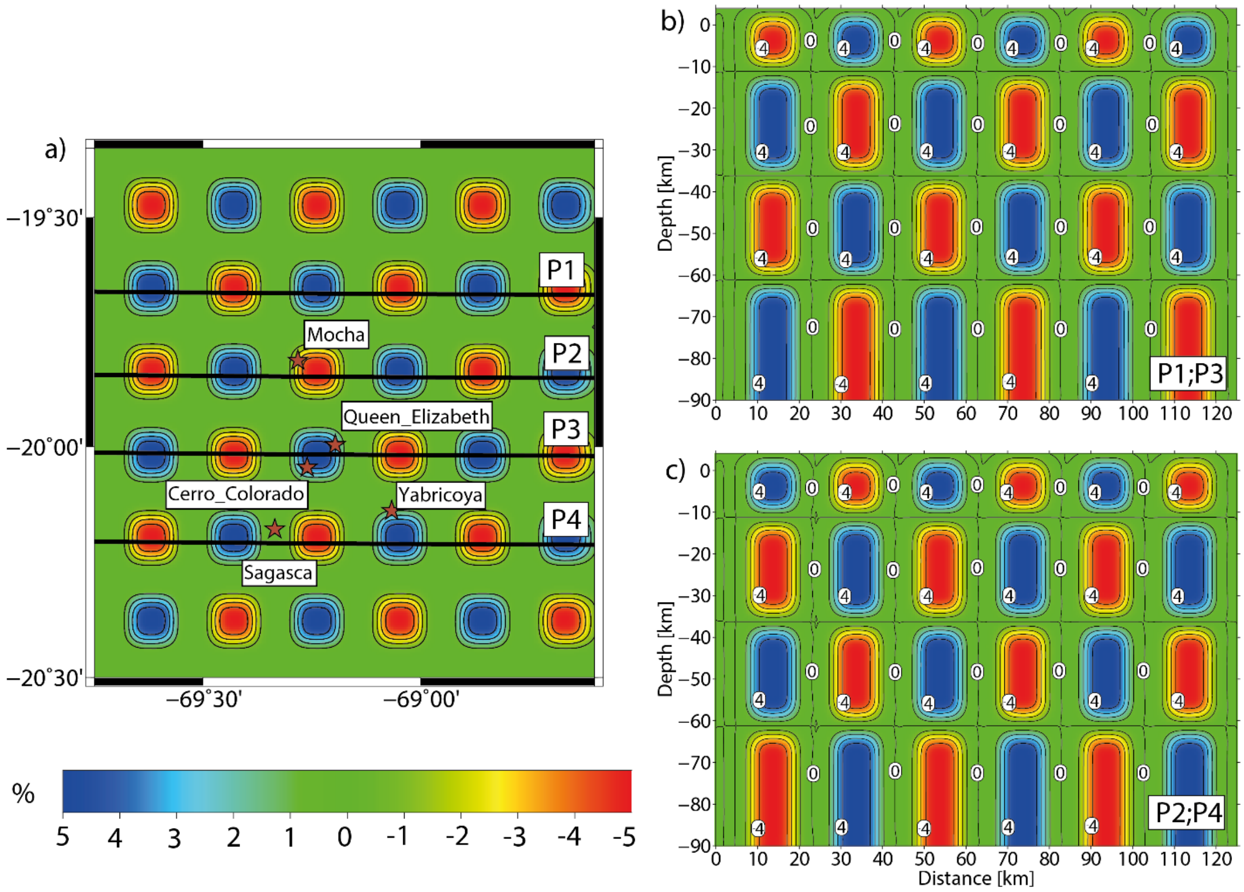

Figure S2. Checkerboard resolution test for the Vp and Vs velocity models. The initial 1D models were perturbed with anomalies of ± 5% in square prisms of 10 × 10 km^2 dimension in latitude and longitude, and variable length in depth, where the first anomaly is 10 km deep, the second and third anomalies are 20 km deep, and the fourth anomaly is 30 km deep. These perturbances start from 0 km depth, and each anomaly is 5 km apart in all 3 directions. **(a)** Plan view of the original anomalies. Black lines represent P1, P2, P3 and P4 profiles shown in Figure S7 and Figure S8. Note that these profiles are not the same as those defines in the main text (Fig. 4). **(b)** Original anomalies for P1 and P3 profiles shown in Figure S5. **(c)** Original anomalies for P2 and P4 profiles shown in Figure S6. All contours represent an interval of ± 1%. Numbers show the contour where the model was not perturbed (0%), and the contours with the minimum and maximum perturbation. Values are indicated by the color palette at the bottom of the figure. The software used to generate this figure was The Generic Mapping Tools (www.generic-mapping-tools.org), and the final editing was done with the software Adobe Illustrator 2022 (www.adobe.com).

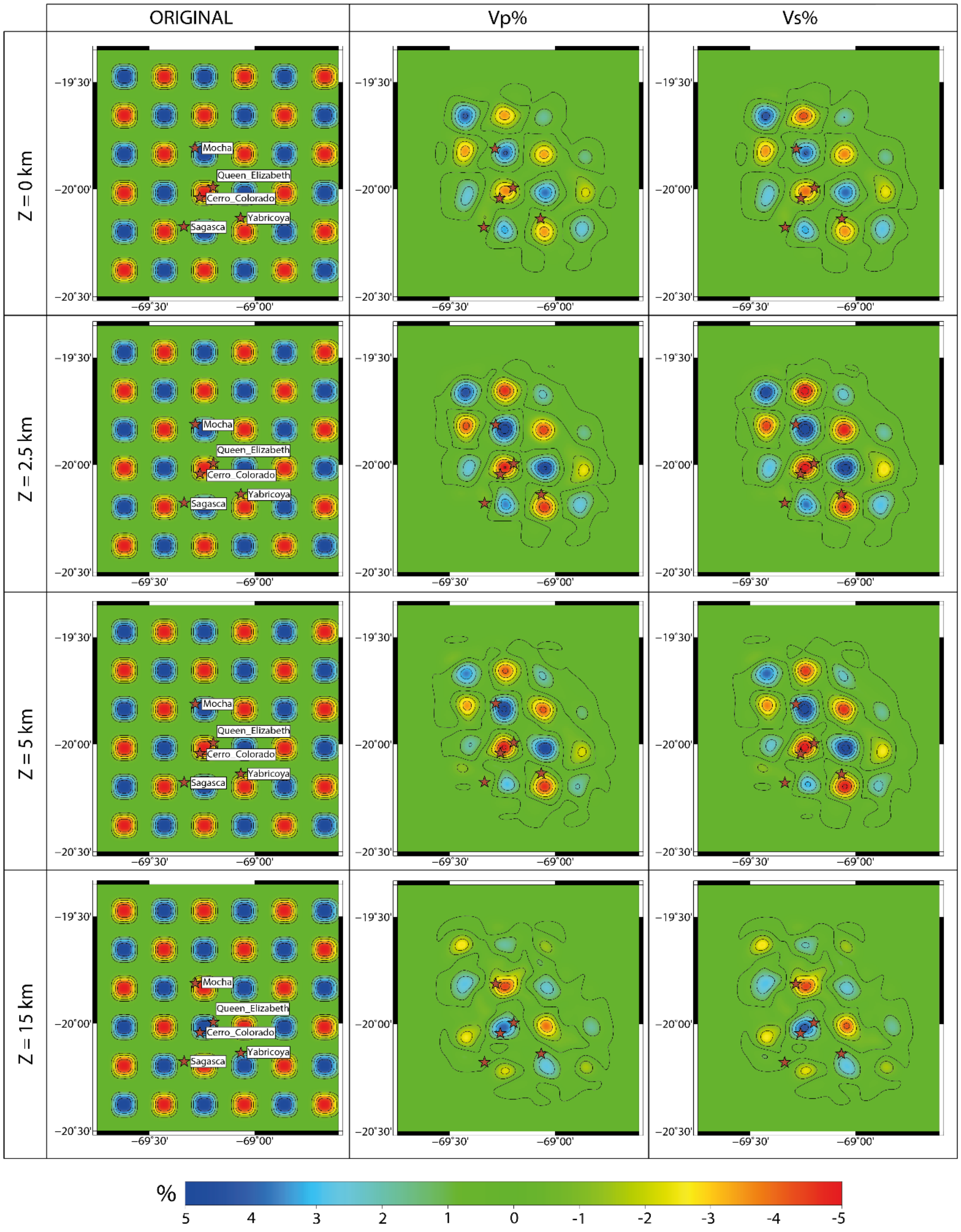

Figure S3. Plan view of results of the checkerboard resolution test at 0 km, 2.5 km, 5 km and 15 km depth. First row corresponds to the original model with the ± 5% perturbations, whereas the second and third rows corresponds to the test result for Vp% and Vs%, respectively. Contours represent an interval of ± 1%. Values are indicated by the color palette at bottom of the figure. Red stars represent main porphyry copper deposits in the study area. The software used to generate this figure was The Generic Mapping Tools (www.generic-mapping-tools.org), and the final editing was done with the software Adobe Illustrator 2022 (www.adobe.com).

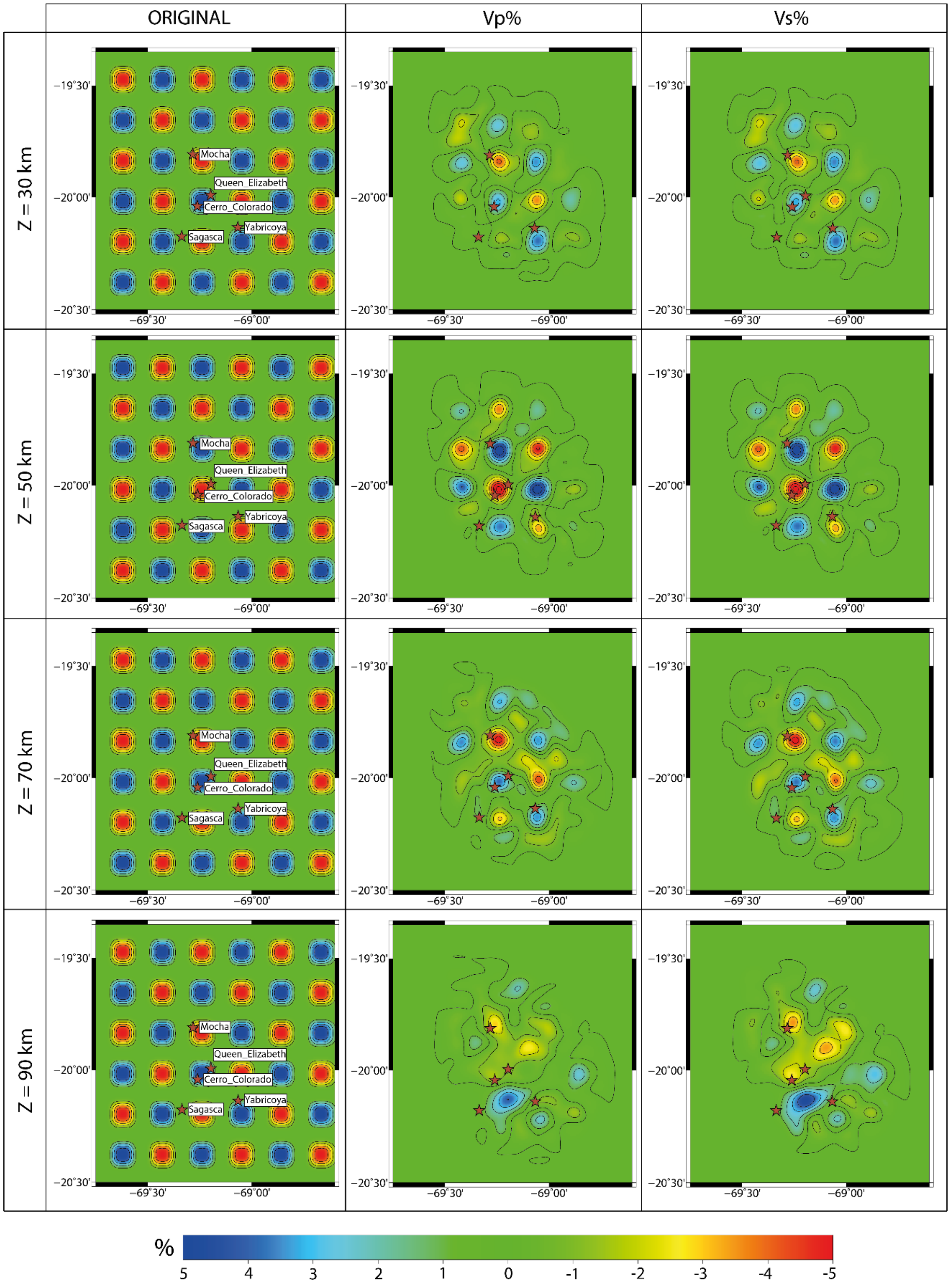

Figure S4. Plan view of results of the checkerboard resolution test at 30 km, 50 km, 70 km and 90 km depth. First row corresponds to the original model with the ± 5% perturbations, whereas the second and third rows corresponds to the test result for Vp% and Vs%, respectively. Contours represent an interval of ± 1%. Values are indicated by the color palette at bottom of the figure. Red stars represent main porphyry

copper deposits in the study area. The software used to generate this figure was The Generic Mapping Tools (www.generic-mapping-tools.org), and the final editing was done with the software Adobe Illustrator 2022 (www.adobe.com).

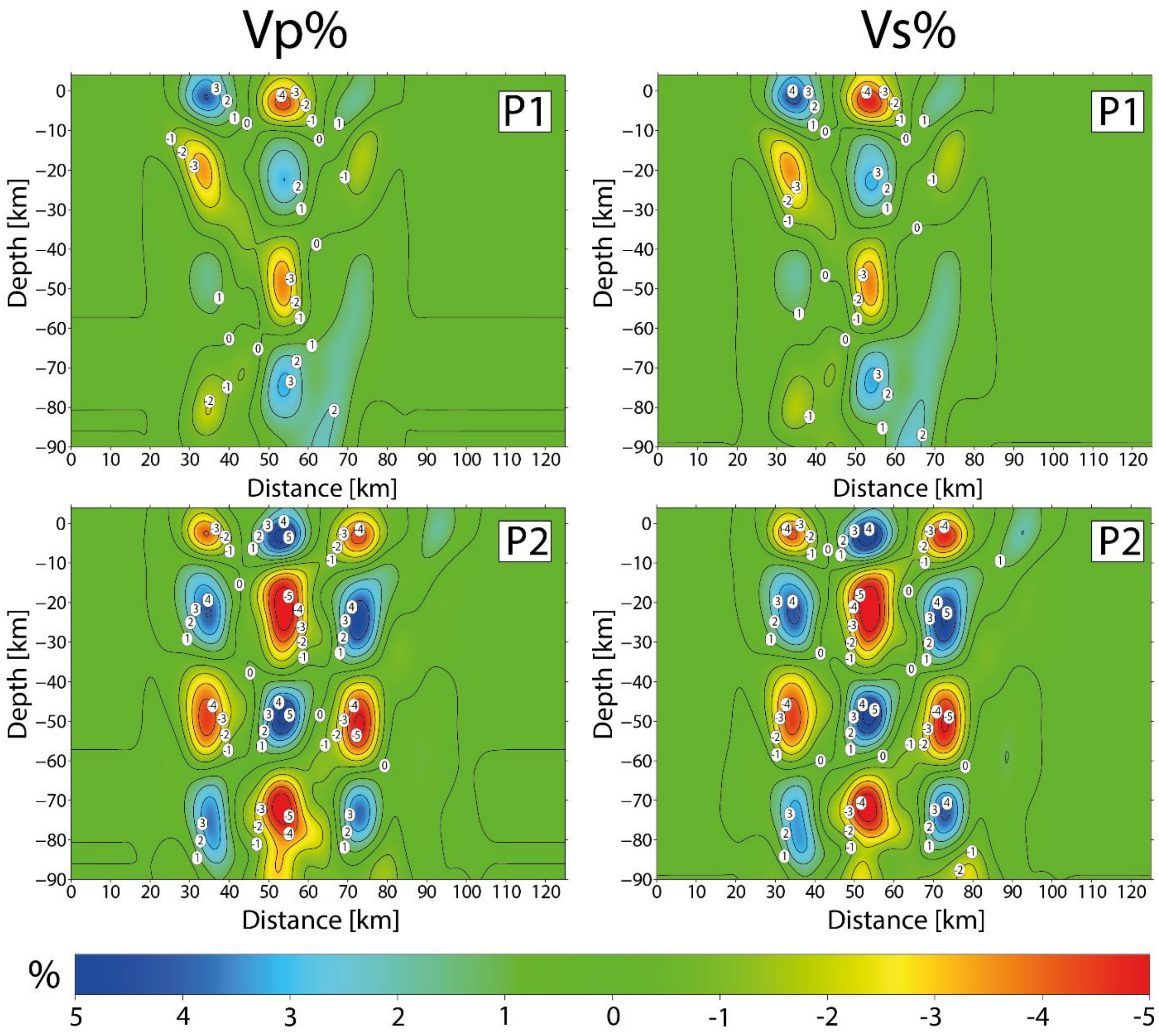

Figure S5. In-depth profiles of the checkerboard resolution test for the Vp and Vs velocity models for P1 and P2 profiles indicated in Figure S2. The left and right columns correspond to the test result for Vp% and Vs%, respectively. Contours represent an interval of ± 1%. Values are indicated by the color palette at the bottom of the figure. The software used to generate this figure was The Generic Mapping Tools (www.generic-mapping-tools.org), and the final editing was done with the software Adobe Illustrator 2022 (www.adobe.com).

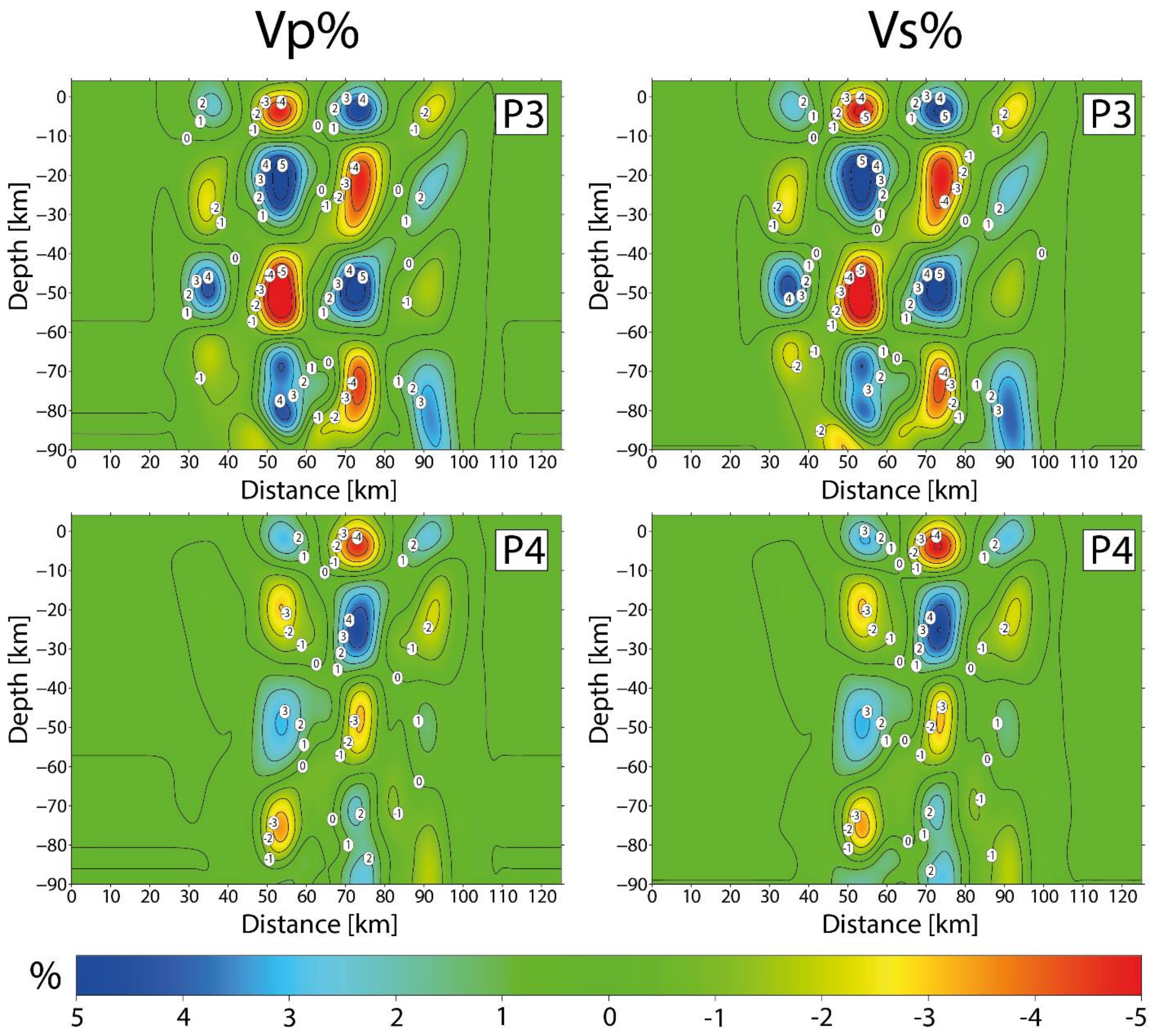

Figure S6. In-depth profiles of the checkerboard resolution test for the Vp and Vs velocity models for P3 and P4 profiles indicated in Figure S2. The left and right columns correspond to the test result for Vp% and Vs%, respectively. Contours represent an interval of ± 1%. Values are indicated by the color palette at the bottom of the figure. The software used to generate this figure was The Generic Mapping Tools (www.generic-mapping-tools.org), and the final editing was done with the software Adobe Illustrator 2022 (www.adobe.com).

Appendix 3. Vp% and Vs% vertical sections along E-W profiles P1-P8

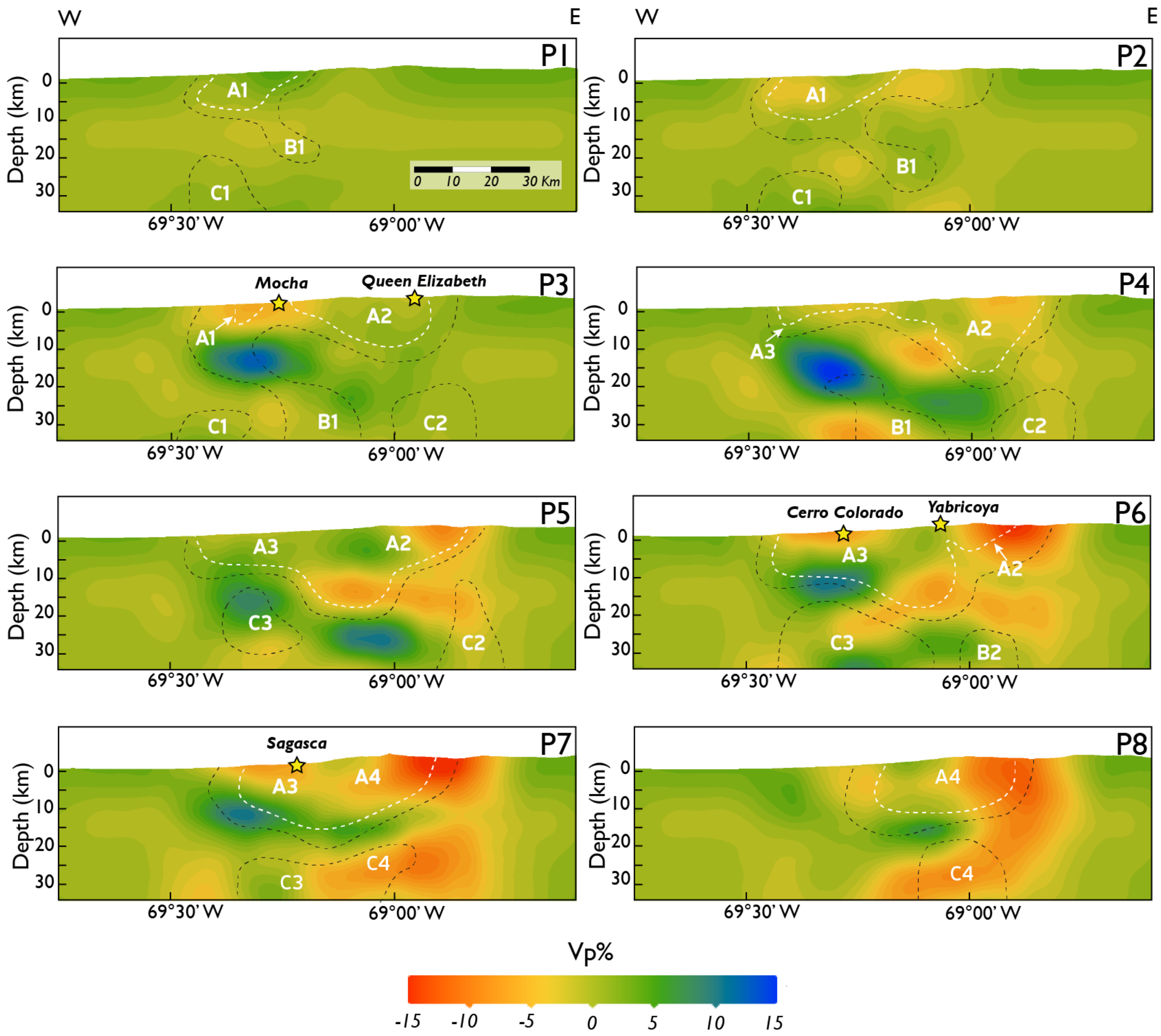

Figure S7. Vertical sections for Vp% along profiles P1-P8 (see Fig. 4 for map location). The white dotted curves denote *low Vp/Vs* areas (see Fig. 5). The yellow stars (surface) correspond to porphyry copper deposits included in the study area. A1-A4, B1-B2, and C1-C4 correspond to the *low*, *medium*, and *high Vp/Vs* anomalies, respectively, described in the main text. The software used to generate this figure was Leapfrog 2022.1 (www.seequent.com), and the final editing was done with the software Adobe Illustrator 2022 (www.adobe.com).

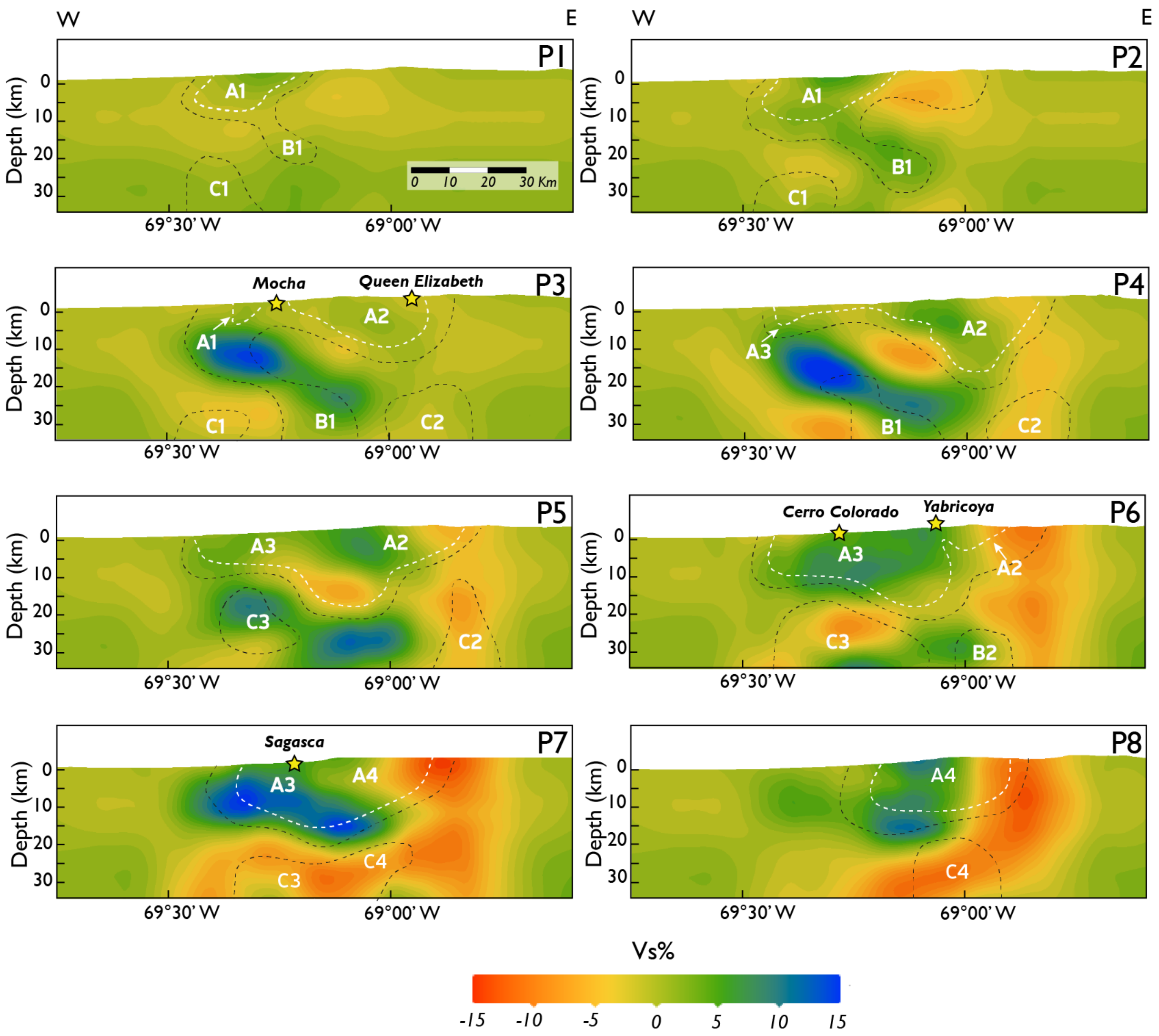

Figure S8. Vertical sections for Vp% along profiles P1-P8 (see Fig. 4 for map location). The white dotted curves denote *low Vp/Vs* areas (see Fig. 5). The yellow stars (surface) correspond to copper porphyry deposits included in the study area. A1-A4, B1-B2, and C1-C4 correspond to the *low*, *medium*, and *high Vp/Vs* anomalies, respectively, described in the main text. The software used to generate this figure was Leapfrog 2022.1 (www.seequent.com), and the final editing was done with the software Adobe Illustrator 2022 (www.adobe.com).

Appendix 4. Three dimensional (3D) Vp/Vs model

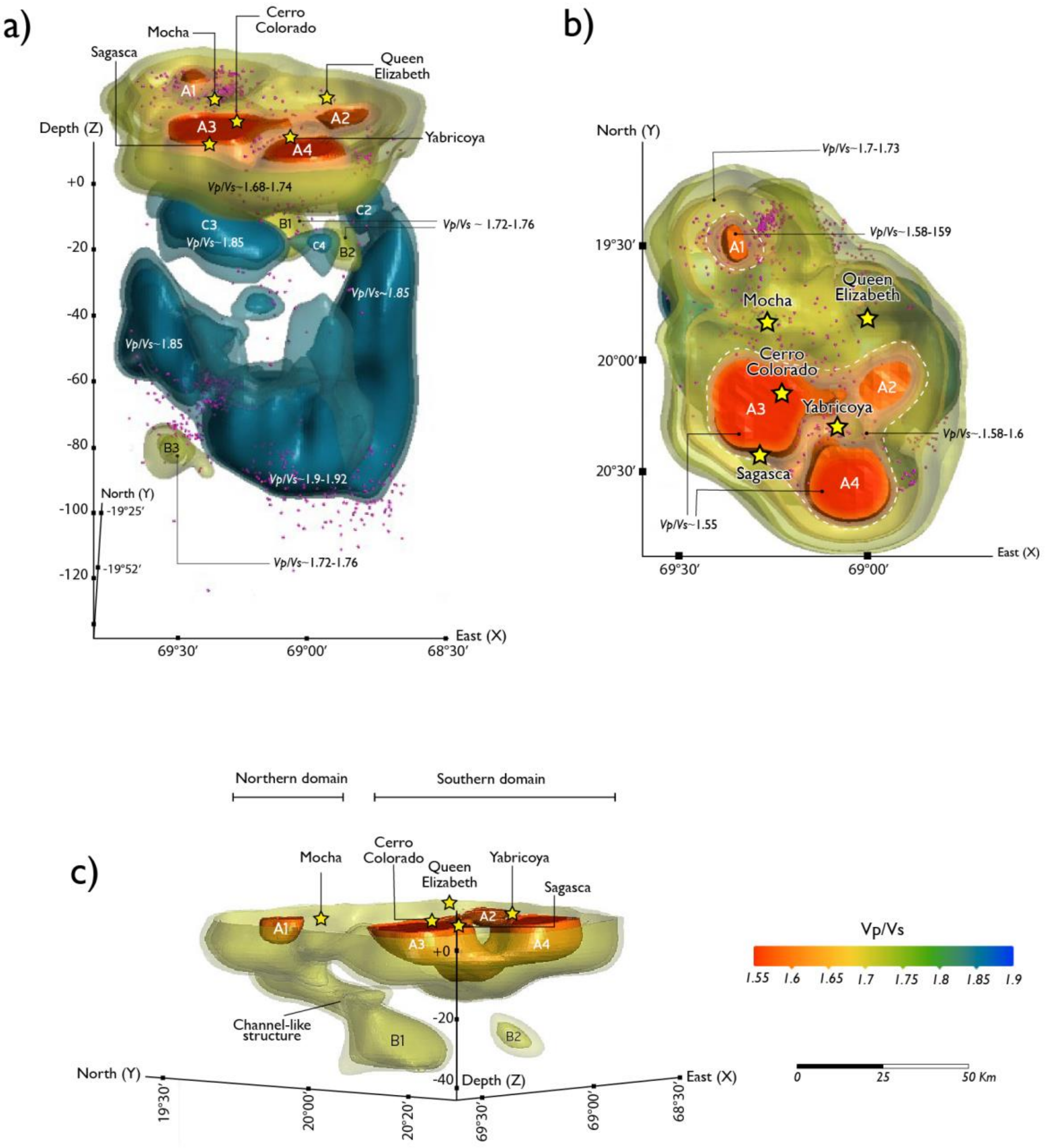

Figure S9. Three-dimensional (3D) Vp/Vs model. **(a)** Perspective view denoting reference values of the Vp/Vs ratios. **(b)** Plan view. **(c)** Shallower *low*-to-*medium Vp/Vs* anomalies (A and B bodies). Yellow stars correspond to known porphyry copper deposits included in the study area. The pink points in figures (a) and (b) correspond to punctual seismicity. See main text for discussion. The software used to generate this figure was Leapfrog 2022.1 (www.seequent.com), and the final editing was done with the software Adobe Illustrator 2022 (www.adobe.com).

Video S1. Conceptual model for the formation of the porphyry copper system beneath the study area based on the distribution of the Vp/Vs ratios. Arrows indicate possible paths of fluids and/or magmas. The dotted lines indicated inferred features and pink circles correspond to the seismicity recorded in the area. We used an average Moho depth from Melnick[52] and Maksymowicz, et al.[53]. The numbers indicated inside the circles correspond to the stages of the porphyry system formation (*Stages 1 to 5*) explained in the main text. See text for discussion. The software used to generate this video was Leapfrog 2022.1 (www.seequent.com), and the final editing was done with the software Adobe After Effects (www.adobe.com).
